# Visualization of *Leishmania tropica* Infection in BALB/c Mice by Bioluminescence Imaging

**DOI:** 10.29252/ibj.24.3.164

**Published:** 2019-12-01

**Authors:** Mahdieh Eskandar, Elham Gholami, Negar Seyed, Yasaman Taslimi, Sima Rafati, Tahereh Taheri

**Affiliations:** Department of Immunotherapy and *Leishmania* Vaccine Research, Pasteur Institute of Iran, Tehran, Iran

**Keywords:** BALB/c mice, Green fluorescent protein, Leishmania tropica, Luciferase

## Abstract

**Background:**

*Leishmania tropica* is the cause of more than one form of leishmaniasis and lacks a known reservoir animal. This study compares the potential infectivity of recombinant and wild-type *L. tropica* in BALB/c mice.

**Methods:**

The potential infectivity of recombinant* L. tropica*^EGFP^ or *L. tropica*^EGFP-LUC^ by two different, the subcutaneous and intradermal, routes was compared using a range of classical detection methods and BLI.

**Results:**

In addition to the results obtained from classical diagnostic approaches, the BLI signals were detected in footpads and ears of *L. tropica-*infected animals. The BLI revealed that a BLI signal can be observed at the inoculation site. The stability of the BLI remained constant in the footpad, but the signal was detectable for only three months in the pinna due to the decline in infection over time.

**Conclusion:**

The presented data are a precise verification of the assumption that BALB/c mice could be used as an experimental model for *L. tropica *infectivity.

## INTRODUCTION

Leishmaniasis is an infectious disease caused by *Leishmania* parasite. Iran is an endemic region for two of the main clinical forms of leishmaniasis, including CL and VL^[^^1^^,^^2^^]^. CL is currently one of the major problems in Iran and other endemic neighboring countries^[^^3^^]^. *Leishmania* is transmitted as either anthroponotic or zoonotic and mainly by *L. tropica* or *L. major*, by order of the prevalence^[^^4^^]^. Some reports have shown that both VL^[^^5^^]^ and mucosal CL can be caused by *L. tropica*^[^^6^^]^. 

BALB/c mice have been used for biological studies against several *Leishmania* species, such as *L. major*, while for *L. tropica,* there are no available mice models^[^^7^^]^. Different rodents, including BALB/c^[^^8^^]^ and C57BL/6^[^^9^^]^ mice, hamsters^[^^10^^]^, rats^[^^11^^]^, and hyraxes^[^^12^^]^ have been applied to study these parasites. 

There are no side effect-free drugs or effective vaccines against leishmaniasis. The main reasons for this deficiency are the lack of a sensitive, specific and rapid test to estimate the extent of parasitic infection and also the absence of an overall pattern of study for all *Leishmania* species. Today, the diagnosis of *Leishmania* is mostly based on assessments of lesion size, culture, and specific staining of the parasite, as well as molecular methods. For many reasons, such as the need to kill many animals, the low sensitivity methods for detecting small numbers of parasites, the time-consuming tests, the unequal distribution of the parasites among different tissues and cells, animal to animal variations, and the impossibility of following the disease process *in vivo*, the quantity of parasites within cells cannot be accurately detected^[^^8^^,^^9^^,^^13^^]^. 

To facilitate the *in vitro* study and detection of leishmanial infection in small animals, reporter genes have been introduced into the *Leishmania* parasite^[^^14^^,^^15^^]^. In particular, BLI shows a linear correlation between the number of parasites and luciferase activity in *Leishmania*^[^^16^^]^.

Each reporter gene, however, indicates some limitations in application such as sensitivity, stability, non-specific background responses, and need for other components that might be difficult or expensive to procure^[^^17^^]^. In order to rapidly identify the presence of this parasite, the infectivity potential of three lines of *L. tropica* including *L. tropica*^WT^, *L. tropica*^EGFP^ (expressing enhanced green fluorescent protein) and *L. tropica*^EGFP-LUC ^(expressing EGFP and luciferase)^[^^18^^]^ were compared in BALB/c mice using routine methods. Also, two recombinant *L. tropica* lines (*L. tropica*^EGFP ^and *L. tropica*^EGFP-LUC^) were used to track the parasite through the expression of the reporter genes. 

## MATERIALS AND METHODS

**Animals**

Female BALB/c mice (6-8 weeks old, procured from the Pasteur Institute of Iran, Tehran) were selected as the animal models for *L. tropica *due to their easier availability. The BALB/c mice were kept in plastic cages with 12/12 hours in the light-dark cycle.

**Parasite culture**

To maintain the infectivity potential of parasites, the wild-type* L. tropica*^WT^ (MOHM/IR/09/Khamesipour, Mashhad) and two recombinant lines (*L. tropica*^EGFP^ and *L. tropica*^EGFP-LUC^) were passaged in BALB/c mice at least two times before the experiment. The promastigote form of the parasites was cultured and propagated in the complete liquid M199 medium (Sigma, Germany) supplemented with heat-inactivated fetal calf serum 20% (Gibco, Germany) and 40 mM of HEPES, 1 mM of L-glutamine, 0.1 mM of adenosine, 0.5 µg/ml of hemin, and 100 µg/ml of gentamicin (all from Sigma) at 26 °C. For the *in vivo* infection, metacyclic stationary-phase promastigotes were isolated by Ficoll gradient type 400 (Sigma)^[^^19^^]^. For visualization of EGFP expression through fluorescent microscope, the log phase of promastigotes was used. 

**Western blotting**

To confirm the reporter protein expression, Western blotting was used according to the described method elsewhere^[^^20^^]^. Briefly, the whole cell lysate of parasites was electrophoresed on 12.5% SDS-PAGE and transferred onto nitrocellulose membranes (Protean, Schleicher & Schuell, Germany). After blocking, the membranes were incubated with 1:6000 anti-GFP-HRP (*horseradish peroxidase,* Acris Antibodies GmbH, Germany) or anti-LUC-HRP monoclonal antibodies (Acris antibodies GmbH) in blocking solution for 2 h. After removing the extra antibody by washing the membrane and incubating in DAB(3,3'-diaminobenzidine) solution as a substrate, the reacting bands were visualized.

**Animal infection**

Mice were infected intradermally in the left ear or subcutaneously in the left footpad with metacyclic promastigotes (~10^7^ p/mice). The footpad thickness was monitored by a digital caliper (resolution: 0.01 mm) every two weeks. At the same time, the induration diameter of ear redness and inflammation was monitored and measured by a ruler.

**Luciferase activity measurements**

The viable promastigote parasites were washed with PBS, and 50 ml of the parasite suspension was serially diluted 1:2 with 50 µl of Glo lysis buffer (Promega, USA) in 96-well black plates at room temperature. After 5 min, equal volumes of luciferin (Promega) were added to each well as substrate. The luminescence intensity was measured using a luminometer reader (Multimode Microplate Reader, Synergy, BioTech Instruments, USA), and the light reaction of each well was measured for one second as relative luminescence units.

**Parasite burden estimation**

At different time periods (1, 2, 3, and 6 months after infection), popliteal or retromaxillar LNs were isolated from 4-5 mice in each group, separately homogenized and serially diluted in Schneider’s Drosophila medium (Sigma) containing heat-inactivated fetal calf serum 20% and gentamycin (100 mg/ml) by a dilution factor of 5 (1:5, from 10^−1^ to 10^−20^). Each dilution was then transferred to 96-well microtitration flat bottom plates in duplicates (150 µL/well). Parasite growth and multiplication were monitored daily by microscopic observation for two weeks, and parasite burden was calculated by the following formula^[^^21^^]^: −log_10_ (last dilution/weight of LN).

***In vivo*** **imaging**

For the *in vivo* bioluminescence, 15 mg/ml of D-luciferin potassium salt (Caliper Life Science, USA) in calcium- and magnesium-free PBS was injected intraperitoneally (*at a* dose of 150 *mg/kg*) as a substrate. Five minutes later, the mice were intraperitoneally anaesthetized with ketamine (10%) and xylazine (2%) injections. The *in vivo* imaging was performed using KODAK imaging system (system FX Pro) with three different modes (luciferase, white, and GFP modes) and different exposure times (15-20 min, 1 s, and 30 s, respectively). The rainbow color images were then placed to overlap the black and white images. After the regions of interest selection, the number of pixels/regions of interest was counted using Molecular Imaging V.5.0.1.27 Software to quantify the light level.

**Statistical analysis**

All the data were presented as mean ± SD, and the statistical comparison of the two data sets was carried out by a positive comparison through the student’s *t*-test using GraphPad Prism Software version 6 for windows (La Jolla, California, USA).

**Ethical statement**

The above-mentioned sampling protocols were approved by the Research Ethics Committee of Pasteur Institute of Iran, Tehran (ethical code: IR.RII.REC. 1395.32).

## RESULTS

**Expression of EGFP in** ***L. tropica*** ^EGFP^ **and** ***L. tropica*** ^EGFP-LUC^

**Fig. 1:**
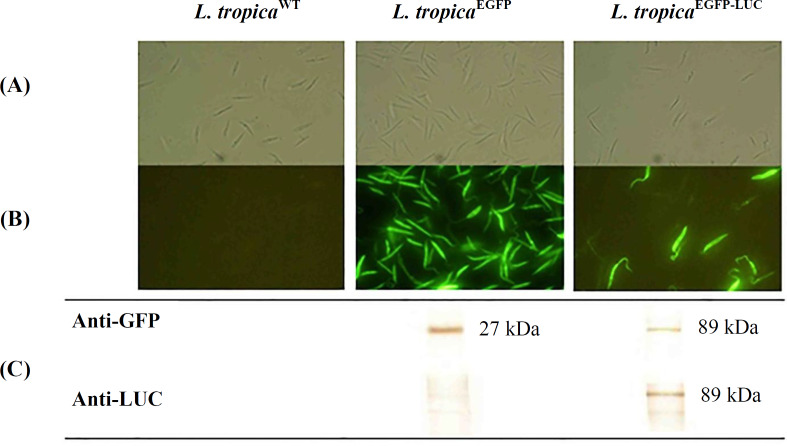
Recombinant parasite confirmation. (A) Light microscopy images, (B) fluorescent microscope images, and (C) Western blot analysis. Expression of EGFP-LUC protein (~89 kDa) was confirmed in *L. tropica*^EGFP-LUC^ parasite using both antibodies (anti-GFP and anti-LUC), and expression of EGFP protein (~27 kDa) was also confirmed in *L. tropica*^EGFP^ through anti-GFP

Previously, to generate two recombinant lines of *L. tropica* expressing EGFP or EGFP-LUC, a linear DNA fragment containing EGFP^[^^14^^]^ or EGFP-LUC^[^^20^^]^ adjacent to SSU regions (small subunit of ribosomal RNA gene) was inserted into the locus of the 18srRNA of the *L. tropica *genome^[^^18^^]^. The two transfected lines of parasites were phenotypically analyzed through light and fluorescent microscopes (Fig. 1A and 1B). After genetic confirmation by PCR^[^^18^^]^, the phenotypic validation of the recombinant parasites was performed via Western blotting using anti-GFP and anti-LUC antibodies (Fig. 1C).

**Monitoring the level of infectivity in BALB/c mice**

**Fig. 2:**
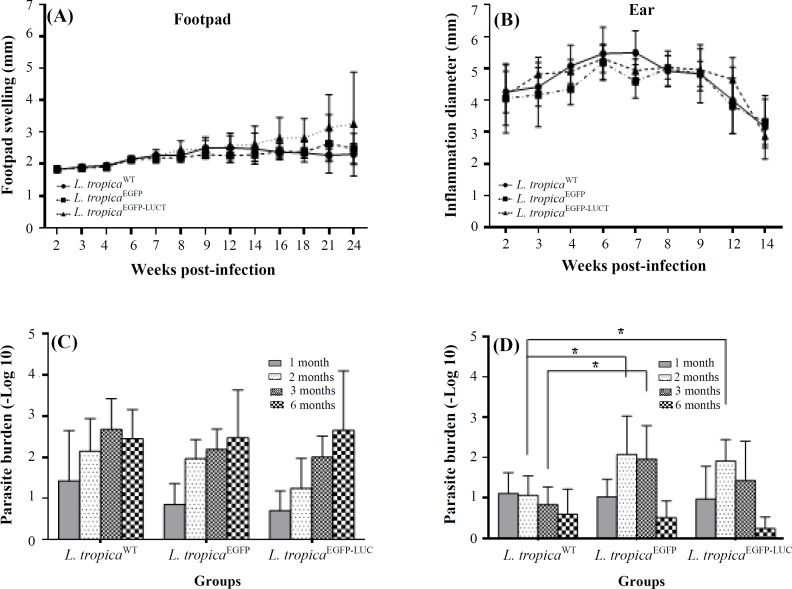
Monitoring the spread of the disease and the measurement of parasite burden in the footpad and ear injected with different lines of *L. tropica* (*L. tropica*^WT^, *L. tropica*^EGFP^, and *L. tropica*^EGFP-LUC^). (A) Measuring the infected footpad’s thickness by a caliper in groups G1, G2, and G3; (B) measuring the diameter of inflammation in the infected ear by a ruler in G4, G5, and G6; (C) parasite load in the popliteal dLNs in G1, G2, and G3; (D) parasite burden in the retromaxillar dLNs in G4, G5, and G6 assessed through the limiting dilution method at four time points (1, 2, 3, and 6 months after the start of the infection). The bars indicate the SD of the mice or dLNs (4-5 individual mice in each group). ^*^p < 0.05

To choose a suitable location to track infection through imaging, two different routes were used for parasite inoculation. The exact same number of parasites was injected subcutaneously into the left footpad (groups 1, 2, and 3) or intradermally into the left ear (groups 4, 5, and 6) of the mice. While groups 1 (G1) and 4 (G4) were received wild-type parasite, groups 2 (G2) and 5 (G5) were challenged with *L. tropica*^EGFP^, and groups 3 (G3) and 6 (G6) were infected with *L. tropica*^EGFP-LUC^. At weekly intervals post-infection, the mice infected in the footpad were monitored for any swelling or inflammation at the injection site. In all three groups infected by different lines of *L. tropica*, increasing the thickness of the infected footpad began at the 6^th^ week and progressed thereafter. A mild increase was measured in the footpad infected with all three lines of parasites (Fig. 2A). The mice infected with *L. tropica*^EGFP-LUC^ (G3) were found to experience slightly more thickness in footpad after week 16 (2.807 ± 0.646 mm in G3, 2.383 ± 0.254 mm in G2, and 2.369 ± 0.162 mm in G1), suggesting a non-significant difference. In the groups infected with the recombinant parasites, an increased thickness was observed that gradually augmented until the 24^th^ week. The largest difference in the footpad thickness between the infected groups was observed in the 24^th^ week (2.307 ± 0.335 mm in G1, 2.483 ± 0.47 mm in G2, and 3.25 ± 1.625 mm in G3). In the group that received the *L. tropica* wild type (G1), there was no or only a slight amount of footpad thickness. To show the biological effect in the infected ear, the inflamed area was measured by a ruler. All three parasites generated a slight inflammation in the infected ear up to six weeks post-challenge, and the inflammation resolved completely after this period (Fig. 2B). A minor thickness was recorded for a short time in the infected ear, which was also resolved for all three lines.

**Parasite burden measurement in dLNs**

Two routes of infection among the three lines of parasites were compared in terms of the number of parasites over six months. At four different time points, including 1, 2, 3, and 6 months after infection, dLNs from regions closest to the infected sites in the footpad (popliteal) or ear (retromaxillar) were used for estimating the parasite burden through a limiting dilution assay. The results showed that one and two month(s) after infection, the number of parasites increased in the dLNs of both infected footpads and ears. The infectivity pattern was dissimilar between the two infected sites after two months. Three months post-infection, the number of parasites started to decline in the ear. A direct and simultaneous comparison between the dLNs isolated from the footpad and ear revealed more parasites in the popliteal dLN three months after infection (Fig. 2C). After six months, more parasites were counted in the footpad. Also, according to the measurements of the footpad, the parasite burden in all three infected groups in the footpad showed an increase in the number of parasites in the dLNs over the following six months. The changes in the degree of inflammation in the infected ear showed that the number of parasites decreased after three months and were estimated to be at their lowest by the sixth month (Fig. 2D). In the infected ear, the pattern of disease and inflammation size were exactly the same for all three lines of parasites. Swelling started in the third week after infection and decreased after the eighth week. The parasite burden in the infected ear groups increased gradually and then decreased three months after infection.

**Fig. 3:**
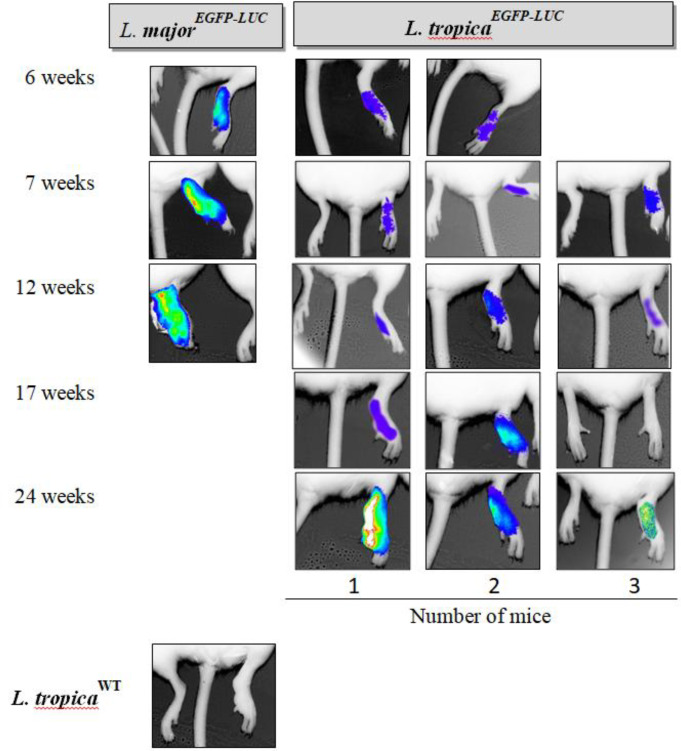
Progressive monitoring of the footpad through BLI in one mouse infected with *L. major*^EGFP-LUC ^(left) as a positive control and two (at week 6^th^) or three mice infected with *L. tropica*^EGFP-LUC^ (right) at different time points, i.e. 6, 7, 12, 17, and 24 weeks after parasite inoculation in the footpad. The first bioluminescence signal-expressing parasite (*L. tropica*^EGFP-LUC^) infection was observed six weeks post-infection, and infection increased with every passing week. The highest signal or rainbow image was apparent 24 weeks after infection. Red and blue areas represent the most and the least intense signals, respectively. In the bottom, the BLI of *L. tropica*^WT^-infected BALB/c mice at 17^th^ week, as a negative control, has been shown

**Progressive monitoring through BLI** ***in vivo***

**Fig. 4:**
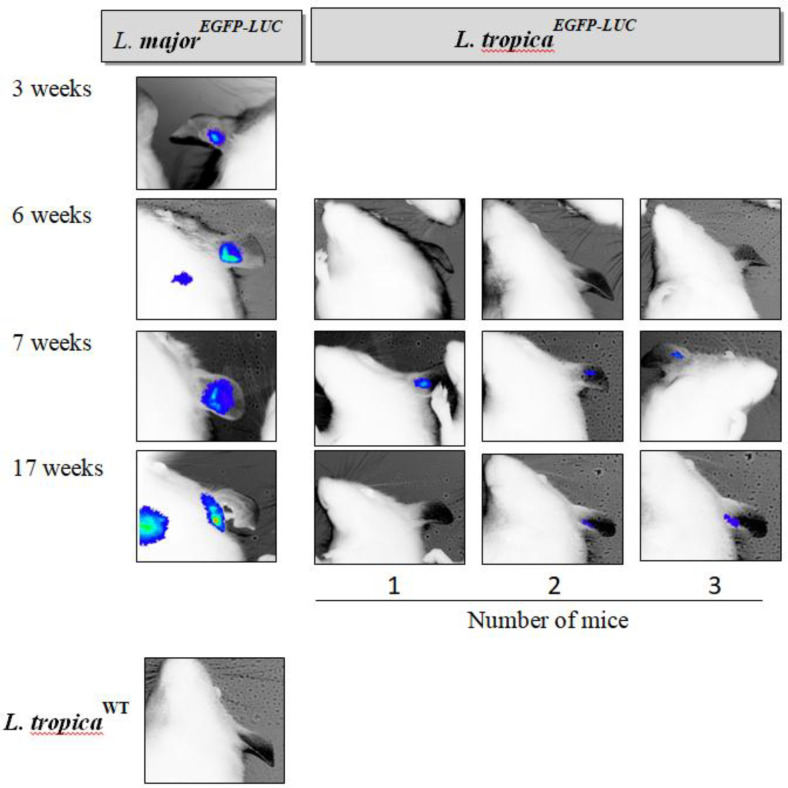
The follow-up of parasite infection in the infected ear through BLI in one mouse infected with *L. major*^EGFP-LUC^ (left) and three mice infected with *L. tropica*^EGFP-LUC ^(right) at 3, 6, 7, and 17 weeks after parasite inoculation. The first bioluminescence signal was observed three weeks after the injection of *L. major*^EGFP-LUC ^that was used as a positive control and seven weeks after the inoculation of *L. tropica*^EGFP-LUC^ in the ear. The highest intensity in the infected mice by recombinant *L. tropica* was detected at the seventh week. Next time (at the 17^th^ week), the intensity of bioluminescence decreased. Regarding the mice infected with recombinant *L. major*^EGFP-LUC^, the first sign of infection was detected at week three, and the strongest signal of BLI was observed 17 weeks after the injection of the parasite. At the 17^th^ week, infectivity level was detected even in the LNs. Red and blue areas represent the most and least intense signals, respectively. In the bottom, the BLI of *L. tropica*^WT^-infected BALB/c mice at 17^th^ week, as a negative control, has been shown

**Fig. 5.**
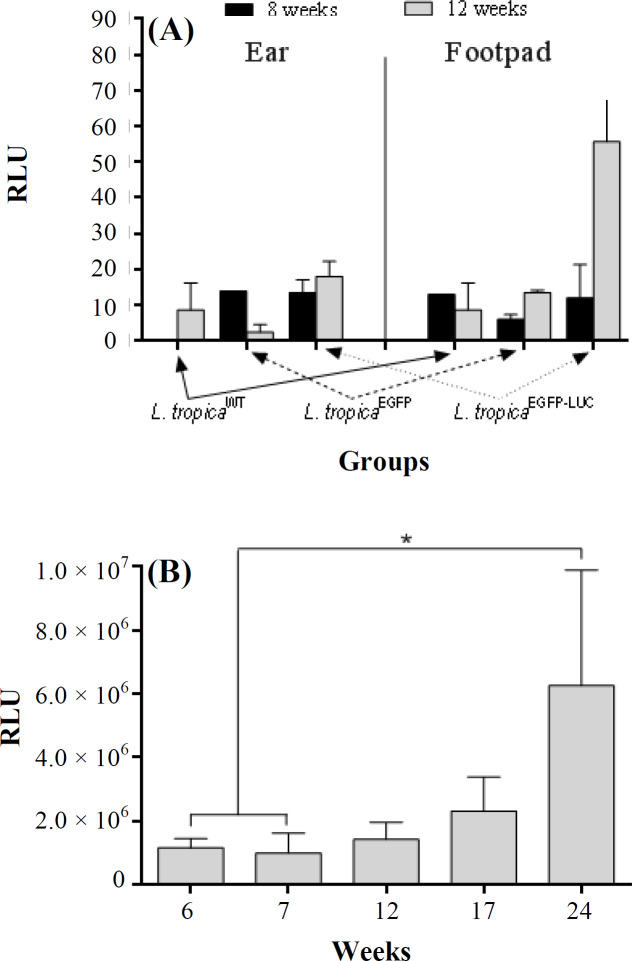
The quantification of LUC expression in the dLNs of BALB/c mice infected with *L. tropica*^EGFP-LUC^ in two different conditions, *in vitro* and *in vivo*. (A) Mice were infected with recombinant *L. tropica*^EGFP-LUC^, and the dLNs were isolated from four mice and were homogenized individually in lysis solution 8 and 12 weeks after the injection of the parasite in the footpad. After 5 min, D-luciferin was added to each well as a substrate. Enzymatic reaction was quantified by 1 s/well and a sensitivity of 100 immediately thereafter; (B) the regions of interest from the footpad of the mice infected with *L. tropica*^EGFP-LUC^ were quantified in pixels and calculated by the sum of intensity (mean ± SD) at different time points. Data are shown as the mean ± SD of the LUC activity of each dilution in duplicate

The main aim of this study was to use *in vivo* imaging technology to evaluate disease progression in BALB/c mice infected subcutaneously and intradermally with two different lines of recombinant *L. tropica*. For this purpose, mice infected with *L. major*^EGFP-LUC[^^20^^]^ in both the footpad and ear were studied as the positive controls. At first, no signals of the expression of reporter proteins–such as that observed in mice infected with *L. major*^EGFP-LUC[^^20^^]^ were observed in the footpad or LNs infected with *L. tropica*^EGFP-LUC^. Different conditions, such as increase in the concentration of luciferin and longer exposure times, had to be tested. To visualize the luciferase signal in live mice infected with *L. tropica*^EGFP-LUC^, the time of exposure had to be increased by about 15-20 minutes in comparison with the mice infected with *L. major*^EGFP-LUC^, for which this time was about 10 minutes^[^^20^^]^. After optimizing the time, whole body imaging of the live mice was carried out through *in vivo* bioluminescence and fluorescence without sacrificing the animals. The first bioluminescence signal was observed at around 6 and 7 *weeks* after the injection of *L. tropica*^EGFP-LUC^ in the footpad and ear, respectively (Figs. 3 and 4). The minimum duration for monitoring the LUC signal in the mice infected with recombinant *L. major*^EGFP-LUC^ was less than 2 and 3 weeks in the footpad^[^^20^^]^ and ear (Fig. 3), respectively. Footpad BLI and the measurement of the sum of intensity also showed that the bioluminescence signal was measurable and actually increased during the study. Besides, the highest luciferase intensity was measured 24 weeks after infection in the footpad (Fig. 3). The highest level of infection in the footpad was detected at the late stage of infection, which is after 24 weeks (in this study), using bioluminescence (Fig. 5B). In the infected ear, the first bioluminescence signal was recognizable seven weeks after infection in all three infected mice. The BLI signal decreased after two months and was measurable after 17 weeks. In the ear infected with *L. major*^EGFP-LUC^, as the positive control, the BLI signal could be observed from the third week and increased until the end of the experiment. The BLI signal was observed even in the LN at this time (Fig. 4). Luciferase activity in the homogenized dLNs from all three groups was compared between the LUC expression (*L. tropica*^EGFP-LUC^) and no expression groups (*L. tropica*^WT^ and *L. tropica*^EGFP^), which were also used as the negative controls, at two time points, including 8 and 12 weeks after infection. Luciferase activity in the lysates of dLNs (*in vitro*) showed that the highest relative luminescence units activity was measured 12 weeks after infection, as compared to the earlier time point (eight weeks) and also to the control groups (*L. tropica*^WT^ and *L. tropica*^EGFP^). A special increase was shown in BLI in the infected footpad in comparison with the infected ear (Fig. 5A). This observation proved the expression and activity of LUC in the infected tissue. During this period, no infections were observed by any lines of *L. tropica* in other tissues, especially the LNs of the BALB/c mice, through imaging (Figs. 3 and 4). In addition, the infection rate and parasite burden were quantified by measuring the sum of the intensity of the bioluminescence signals obtained after the *in vivo* imaging of the footpads at different time points post-infection (Fig. 5B). Both the BLI images (Fig. 3) and the sum of the infectivity rate (Fig. 5B) clearly confirmed that the infection level had increased in the footpad gradually until 24 months post-infection. Furthermore, no EGFP signal was observed in the footpads or ears infected with recombinant *L. tropica* parasite (*L. tropica*^EGFP^). No EGFP signal was detected in the mice infected with *L. tropica*^EGFP-LUC^. A direct comparison between the infected mice through imaging showed that in spite of applying a larger number of *L. tropica* (10^7^ p/mouse) in each tissue compared to *L. major* (10^5^ p/mouse), higher levels of BLI could be detected in the mice infected with *L. major*. Also, no LUC signals were detected in the LNs of mice infected with *L. tropica*^EGFP-LUC^.

## DISCUSSION

Demonstration of CL in infected footpad of BALB/c mice by *L. major* and *L. tropica* is not similar in aspect of inflammation, development of footpad size, and creation of swelling. However, detection of parasite number in the body of mice is very critical to research and therapy study.

In this work, in spite of the similarity between groups 1, 2, and 3, which were infected with three lines of *L. tropica* parasites, footpad thickness in G3 that infected with *L. tropica*^EGFP-LUC ^increased slightly around the 4^th ^month. Besides, the difference in footpad size was not significant among the three groups of mice infected with different lines of *L. tropica*. This result was expected because the expression of the two reporter genes in *L. major * had previously been shown to increase swelling in the footpad^[^^20^^,^^21^^]^. The infectivity development through both routes of infection showed that the expression of the reporter genes did not affect the sensitivity and infectivity potential of the recombinant parasites in detecting parasites in mammals. Nevertheless, measuring footpad/inflamed size remained as a problem, as it is not a very sensitive indicator of the development of the disease and evaluation of infection severity^[^^16^^,^^22^^]^. Also, the difference of parasite load between different groups of mice was insignificant when the parasites were injected subcutaneously in footpad. The number of parasites in dLNs increased during six months post-infection. On the other side, measuring the parasite load showed decreased amount of parasite after almost two months, following intradermal injection of *L. tropica* to ear. Interestingly, these data express that the second month post-infection is roughly the most suitable time to study the ear.

Some differences were observed between two routs of infection. The number of parasites in the retromaxillar dLNs was considerably lower than that of the popliteal LNs. Another difference between the two routes of infection was the disparity in the stability of infectivity in the infected tissue. In the popliteal, the parasite number increased during the study period, but in the retromaxillar, this number decreased after two months. The results, thus, showed that the route of infection is important for parasite estimation. As reported before, the route of infection in an animal model is a very vital subject for generating suitable immunity against leishmaniasis^[^^23^^]^. Furthermore, the BLI method allowed the observation and measurement of transfected parasite load in the real-time of infection in infected footpad and ear. Moreover, we could visualize and follow the infectivity rate in both infected sites. The lack of EGFP signal in recombinant parasite *L. tropica*^EGFP-LUC^ was predictable owing to highly structure-dependent protein, and fusing EGFP with other proteins resulted in a misfolding and a decline in the fluorescence detectability of EGFP^[^^24^^]^. The lack of EGFP fluorescence and expression in mice infected with *L. tropica*^EGFP^ may be due to the reduced number of parasites^[^^22^^]^ or the low expression or low sensitivity of this protein in the tissue.

In a previous study, bioluminescent parasites were observable just one day after inoculation into the ear or footpad of rats (another possible animal model for *L. tropica*) by *in vivo* imaging^[^^11^^]^. These researchers mentioned that the deficiency in the spread of infection to LNs may have been due to the low sensitivity of the method used to study this type of parasite^[^^11^^]^.

The data presented in this study showed that monitoring luciferase expression, which is expressed by transfected parasite, is possible in both tissues, footpad or ear, in mice infected with *L. tropica*^EGFP-LUC^. This finding facilitates the more precise evaluation of the infection level and the growth of parasites *in vivo*. Hence, using LUC-expressing *L. tropica* might increase the sensitivity of parasite detection for BLI studies. However, the difference between the two parasites, *L. major* and *L. tropica*, was primarily because of the differences between the infection patterns of the two *Leishmania* species and also because of the susceptibility to specific parasites in the BALB/c mice^[^^25^^]^.

This investigation is the first report on the use of BLI for BALB/c mice models infected with *L. tropica*. Many questions remain to be answered on this subject because *L. tropica* is an unusual *Leishmania* species that needs more research from different aspects. Nonetheless, due to the lack of sensitive animals for some *Leishmania* species such as *L. tropica* and also the problem of variation between the mice strains^[^^8^^]^, using more than one method is highly recommended. Nevertheless, this study suggests, for the first time, that noninvasive BLI can be helpful for real-time monitoring of infection in animal models, especially during the initial stage of disease caused by *L. tropica* infection.
